# MODIS Sensor Capability to Burned Area Mapping—Assessment of Performance and Improvements Provided by the Latest Standard Products in Boreal Regions

**DOI:** 10.3390/s20185423

**Published:** 2020-09-22

**Authors:** José A. Moreno-Ruiz, José R. García-Lázaro, Manuel Arbelo, Manuel Cantón-Garbín

**Affiliations:** 1 Departamento de Informática, Universidad de Almería, 04120 Almería, Spain; jaruiz@ual.es (J.A.M.-R.); jrgarcia@ual.es (J.R.G.-L.); mcanton@ual.es (M.C.-G.); 2 Departamento de Física, Universidad de La Laguna, 38200 San Cristóbal de La Laguna, Spain

**Keywords:** remote sensing: burned area, wildfire, MODIS, MCD45A1, MCD64A1, fire_CCI, pareto boundary

## Abstract

This paper presents an accuracy assessment of the main global scale Burned Area (BA) products, derived from daily images of the Moderate-Resolution Imaging Spectroradiometer (MODIS) Fire_CCI 5.1 and MCD64A1 C6, as well as the previous versions of both products (Fire_CCI 4.1 and MCD45A1 C5). The exercise was conducted on the boreal region of Alaska during the period 2000–2017. All the BA polygons registered by the Alaska Fire Service were used as reference data. Both new versions doubled the annual BA estimate compared to the previous versions (66% for Fire_CCI 5.1 versus 35% for v4.1, and 63% for MCD64A1 C6 versus 28% for C5), reducing the omission error (OE) by almost one half (39% versus 67% for Fire_CCI and 48% versus 74% for MCD) and slightly increasing the commission error (CE) (7.5% versus 7% for Fire_CCI and 18% versus 7% for MCD). The Fire_CCI 5.1 product (CE = 7.5%, OE = 39%) presented the best results in terms of positional accuracy with respect to MCD64A1 C6 (CE = 18%, OE = 48%). These results suggest that Fire_CCI 5.1 could be suitable for those users who employ BA standard products in geoinformatics analysis techniques for wildfire management, especially in Boreal regions. The Pareto boundary analysis, performed on an annual basis, showed that there is still a potential theoretical capacity to improve the MODIS sensor-based BA algorithms.

## 1. Introduction

Wildfires cause deforestation and habitat loss, and they are responsible for releasing a huge amount of aerosol particles and greenhouse gases into the atmosphere. These emissions vary depending on the Burned Area (BA) extension and on the type of biomass present in the region where the fire occurs. For example, Equatorial Asia, which is responsible for only 0.6% of the Global Burned Area (GBA), generates CO_2_ and CH_4_ emissions of 8% and 23%, respectively. Meanwhile, boreal forests, responsible for 2.5% of GBA, emit 9% of global CO_2_ and 15% of CH_4_ emissions [1]. Annually, between 5 and 15 million ha are burned in boreal forests, mainly in Siberia, Canada and Alaska, and the projections of different climate models estimate from decreases to increases in BA, which, as suggested by Kitzberger et al. [2], generates uncertainty in boreal regions, where global warming may create contrary effects.

An accurate BA estimation is therefore essential for predicting changes in the global climate system, increased greenhouse gas concentrations or the changing chemical composition of the atmosphere due to fire emissions. Detailed spatial and temporal knowledge of BA is also essential in dynamic global vegetation models (DGVM) where, together with other geospatial data, many ecological variables can be quantified and projected. We should also not forget the importance of BA maps, in combination with socioeconomic and meteorological data, in signaling which factors control the recurrence of fire and how long they last at the regional or global level [3].

Since the early 1970s, sensors onboard numerous Earth observation missions, such as AVHRR (Advanced Very High Resolution Radiometer), SPOT-VGT (Satellite Pour l’Observation de la Terre-Vegetation), ATSR (Along Track Scanning Radiometer), MODIS (Moderate Resolution Imaging Spectroradiometer), or Landsat, have made it possible to derive BA products on a global and/or regional scale. Among these products, we can highlight GLOBSCAR [4], GBA2000 [5], GBS (Global Burned Surfaces) [6], GLOBCARBON [7], L3JRC [8], GEOLAND2 [9], Global Fire Emission Database (GFED) [10], BAECV (Burned Area Essential Climate Variable) product [11] and GIO-GL1 (Copernicus Global Land Service burned area product) based on the Tansey et al. algorithm [8]. While some of the above-mentioned products remain operational, the main products currently in use are Fire_CCI 5.1 developed by the ESA [12] and MCD64A1 C6 developed by the University of Maryland [13]. Both BA detection methods are based on reflectance derived from solar reflective bands in combination with thermal anomaly maps from active fires (hotspots) of MODIS [14]. The MODIS sensor has been operational since 2000. It has 36 spectral bands from 0.45 µm to 14.385, a 12-bit radiometric resolution, and spatial resolutions of 250 m, 500 m, and 1 km [15,16].

Various international scientific programs that deal with global fire assessment defined key objectives of spatial and temporal accuracy, and a set of basic features that BA products must fulfill. The work by Mouillot et al. [3] presented the summarized instructions of the Integrated Global Observing Strategy (IGOS) [17,18], Global Terrestrial Observing System (GTOS), the Group on Earth Observations (GEO) Carbon strategy [19], the Global Climate Observing System (GCOS) and the NASA White Paper on Fire Earth System Data Records (Fire ESDR) [20]. On the one hand, long time series (greater than several decades) that are consistent and temporally stable are required to understand the interaction between climate, vegetation, and fire. A spatial resolution of between 250 and 500 m would be desirable. With regard to the spatial accuracy of the products, although some users state that BA products are acceptable when omission and commission errors are balanced [21,22,23], most set a maximum of around 20% for both CE and OE [23,24,25]. Do the Fire_CCI 5.1 and MCD64A1 C6 products meet those requirements? Given the technical limitations of the MODIS sensor, can BA mapping results be improved by modifying the algorithmic strategy? The answers to these questions, as well as quantifying the accuracy of these two products, turns out to be very valuable information for properly managing wildfires and their consequences using geoinformatics analysis techniques [26].

In this paper, we present a detailed study of the temporal and spatial accuracy of both datasets, focusing on the boreal region of Alaska. The previous versions of both products (Fire_CCI 4.1 and MCD45A1 C5) have also been included to analyze the impact of the changes made in the new versions. The Alaskan region was selected for two reasons. First, it is one of the few regions in the world that has an official database with detailed records of the area burned by all fires since 1940. These data were used as a reference set to assess the accuracy of all the products. Second, the scars left by burned areas in a boreal region such as Alaska persist for longer, thus facilitating detection and more accurate mapping [27,28].

The objectives pursued in this work are as follows:

- To assess the spatiotemporal accuracy of each of the annual time series of the burned area products versus the reference data (AFS) for the 2000–2017 period.
- Concerning to temporal accuracy, to calculate the percentages of the annual burned area detected by each product and to analyze the temporal correlation with the reference data.
- In relation to spatial accuracy, to estimate the main metrics derived from the confusion matrix (commission and omission errors) and determine the Pareto Boundary (PB) for the native spatial resolutions of each product from the reference data to separate the errors of each product from the intrinsic errors associated with its spatial resolution.
- To intercompare the spatiotemporal performance of the latest versions of the Fire_CCI 5.1 and MCD64A1 C6 products and to analyze any possible improvements over previous versions (i.e., Fire_CCI 4.1 and MCD45A1 C5.1).
- To quantify the contribution of burned area fragmentation to the classified map errors, linking the area under the annual Pareto boundary curve with the total annual errors of each product to its spatial resolution.

## 2. Materials and Methods

### 2.1. Study Region

**Figure 1:**
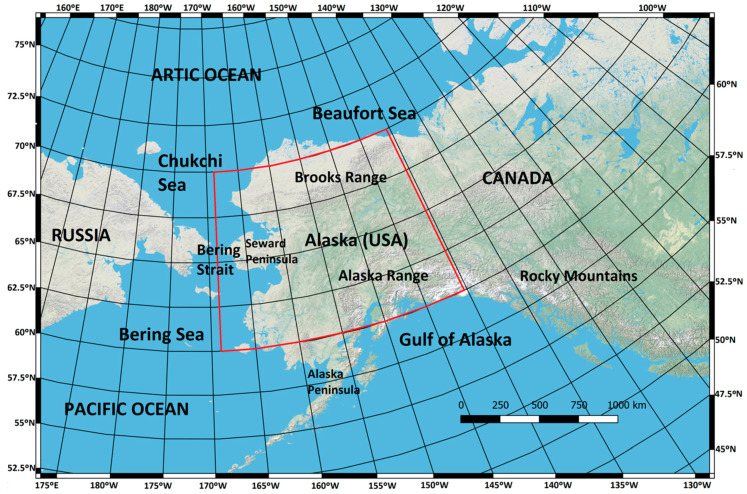
The study region includes the entire boreal forest of Alaska (70° N–60° N, 168.5° W–141° W).

The study area spans a large section of Alaska, extending 10° in latitude (60° N–70° N) and 27.5° in longitude (Figure 1). This area is dominated by boreal forest, a complex set of plant communities modulated mainly by fire, soil type and drainage. The boreal forest forms a mosaic of hardwood–conifer mixed stands with closed canopy in well-drained areas, while in those with permafrost, open spruce stands predominate. Boreal forest makes up 90% of Alaska’s forests, an area of approximately 42 million ha [29].

### 2.2. Reference Data

Polygons delimiting the area burned by wildfires in Alaska are available from the Alaska Fire Service (AFS, Fort Wainwright, AK, USA). AFS has compiled a very complete and accurate database since 1940. In addition to the geographic coordinates of the fire site and perimeter, AFS contains information such as the name of the fire; start and extinction dates; estimated BA; cause (naturally (e.g., lightning), human negligence or maliciously) or municipality of origin. Fire perimeters have always been delineated using the best available data source, from traditional hand-drawing on topographic maps in the early decades, to the interpretation of recent fine-scale satellite images with spatial resolutions less than 30 m [30]. This information was used as the reference data (the ground truth) for the accuracy assessment of the MODIS-derived BA products.

**Figure 2:**
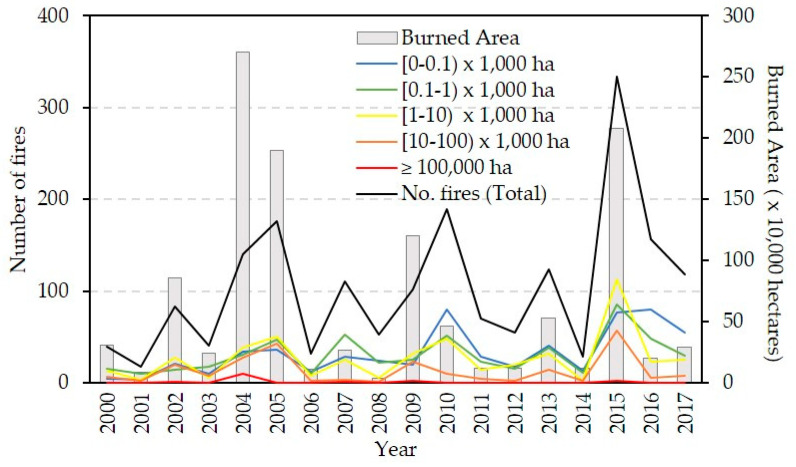
Temporal distribution of the annual burned area in Alaska during the period 2000–2017 and distribution per year of the number of fires by size (in thousands of hectares), according the Alaska Fire Service (AFS, Fort Wainwright, AK, USA). Five categories were considered for the sizes (BA extension in ha) of the fires: very small (<100 ha), small (≥100 ha and <1000 ha), medium (≥1000 ha and <10,000 ha), large (≥10,000 ha and <100,000 ha) and very large (≥100,000 ha).

For the study period, from 2000 to 2017, AFS recorded 1868 fires [31]. The total BA exceeded 11.6 million ha, with an annual average of 0.65 million ha, although strong year-on-year fluctuations were found (see Figure 2): in 12 of the 18 years analyzed, a total BA of 0.5 million ha was not exceeded, with 2001 and 2008 recording the lowest levels, 0.09 and 0.04 million ha, respectively. On the other hand, in 2004 and 2015, the total BA exceeded 2 million ha, with values of 2.71 and 2.08 million ha, respectively.

**Figure 3:**
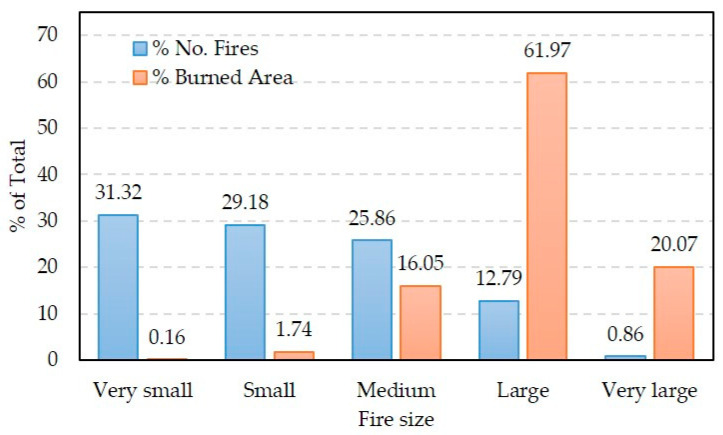
Percentage of the total number of recorded fires by the Alaska Fire Service (AFS) and total area burned according to the five categories considered for the sizes of the fires in Figure 2 for the 18 years of study (2000–2017).

Throughout the period considered, small and very small fires account for an average of 60.50% of the total registered fires, but they only represented 1.90% of the total burned area (Figure 3). In contrast, large and very large fires accounted for 13.60% of the total fires and 82.04% of the total burned area. Between them, the 16 fires of more than 100,000 ha burned 20.07% of the total burned area in the study period (Figure 3).

To construct the annual reference data maps, all the perimeters of fires occurring during the MODIS era (2000–2017) were downloaded from AFS. The annual vector layers were projected to the Albers Conical Equal Area projection using the maximum area method to assign a burned/non-burned class label [32]. The final size of each pixel in the reference maps was 50 m × 50 m.

### 2.3. Burned Area Products

This study compared the main global burned area products using MODIS sensor data: Fire_CCI 5.1 from the ESA project of the same name, led by the University of Alcalá de Henares, and the official NASA MODIS Direct Broadcast Monthly Burned Area Product, developed by the University of Maryland. Both of their most recent versions (Fire_CCI v. 5.1 and MCD64A1 C6), along with the previous versions (Fire_CCI v. 4.1 and MCD45A1 C5.1), were considered to analyze possible performance improvements. Table 1 shows their main characteristics. The main concerns of ESA and NASA when updating their products are to extend the time series and improve the algorithms to obtain the best validation results. Besides, NASA as owner and developer of MODIS sensors periodically reprocesses the entire data archive to incorporate better calibration and improved upstream data into all MODIS products.

**Figure 4:**
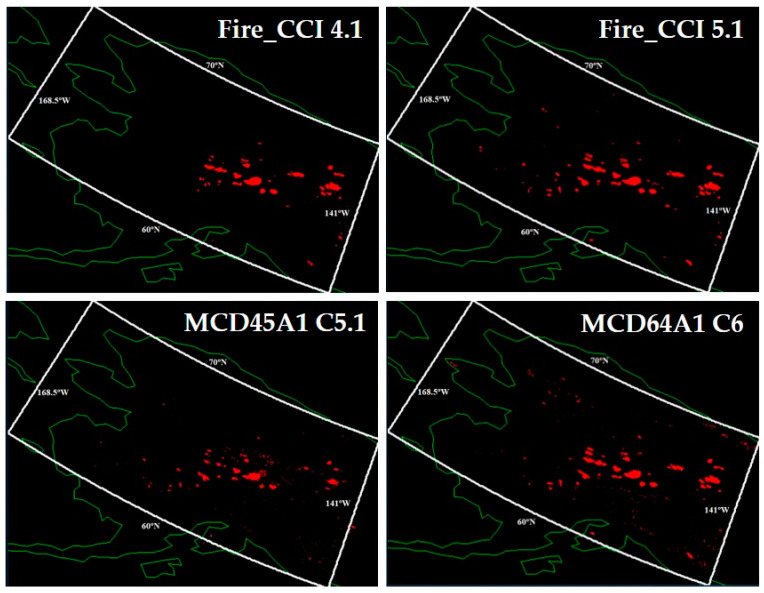
Annual burned area maps for the Alaskan region in 2009. Red, burned pixels; white, study region; green, political borders and coastlines.

To construct the annual BA maps for Alaska, the respective monthly composites of the four products were downloaded. As with the reference maps, all these maps were re-projected to the Albers Conical Equal Area, with a pixel size of 50 m × 50 m, and finally combined on an annual basis. Figure 4 shows the annual maps of each of the products generated for the year 2009.

Three of the four products base their algorithmic strategy on a hybrid approach. They use the MODIS active fires product (hotspots) in combination with changes in daily surface reflectance to identify burned pixels. In contrast, the fourth product (MCD45A1) only uses daily surface reflectance imagery. The following is a brief description of the algorithms used in each of the products.

#### 2.3.1. MCD45A1 Collection 5.1

This BA product uses the MCD45 algorithm to identify burned pixels at a 500 m spatial resolution. It detects changes in the time series of daily bi-directional reflectance in bands 2 (0.841–0.876 μm) and 5 (1.23–1.25 μm) of MODIS sensor [37]. MCD45A1, which is currently deprecated, was the official product until the release of MCD64A1 C6.

The algorithm compares the observed daily reflectance values for each pixel with the predicted values using a bi-directional reflectance model in a 16-day-minimum time window that selects the candidate dates for burning in the forward and backward directions. If both dates match, the pixel is considered burned. That date is then used as a seed to identify, through a contextual iterative process, whether neighboring pixels can be classified as burned or not. In a final step, unselected candidate pixels are considered burned if they have at least three neighbors burned, with their burn date being the average of their neighbors. The algorithm finally excludes those pixels already burned in previous seasons and years [34,35,36].

#### 2.3.2. MCD64A1 Collection 6

This product applies the MCD64 algorithm to identify burned pixels using 500 m daily surface reflectance products for bands 5 (ρ5: 1.23–1.25 μm) and 7 (ρ7: 2.105–2.155 μm), along with daily active fire products (hotspots), both derived from the imaging products of the MODIS sensor on board the Terra and Aqua satellites [38].

The algorithm initially calculates the maximum daily changes in the time series of a burn-sensitive vegetation index VI = (ρ5 − ρ7)/(ρ5 + ρ7) for two pre- and post-date temporal windows. From these dates, it assigns the burn/unburn label to the pixel using the MODIS daily active fire product. It then extracts a set of training samples and performs an initial supervised classification of all pixels based on the normalized distance measurement of each pixel to the nearest pixel in the training set. The final classification is obtained by using contextual information (nearest neighbors) [13].

#### 2.3.3. Fire_CCI 4.1

The Fire_CCI 4.1 product, which has now been discontinued, covers only the period from 2005 to 2011. It identifies burned pixels at a 300 m spatial resolution using time series of daily surface reflectances from the MERIS sensor on board the ENVISAT satellite, and the active fires product derived from MODIS sensor images. The algorithm initially constructs monthly composites of surface reflectances by selecting the candidate pixels to be burned using the MODIS hotspot dates as criteria. It calculates cumulative distribution functions to discriminate the most clearly burned pixels by means of near-infrared reflectance thresholds. Then, it selects seed pixels in a 5 × 5 pixel window centered on the hotspot, to grow the burned regions by contextual analysis of the neighboring pixels. A final filter removes isolated pixels, both burned and non-burned [33].

#### 2.3.4. Fire_CCI 5.1

This is the latest version of the burned area product from the Fire_CCI project. The algorithm, similar to its predecessor, is based on a two-stage hybrid approach. In the first stage, the seed pixels are selected, guided by the daily active fires (thermal anomalies) derived from the MODIS sensors on board the Terra and Aqua satellites. In the second stage, the growth and delimitation of the burned area is performed using the daily surface reflectance products from MODIS sensor bands 1 (0.62–0.67 μm) and 2 (0.841–0.876 μm) at a 250 m spatial resolution [12].

### 2.4. Accuracy Assessment

**Figure 5:**
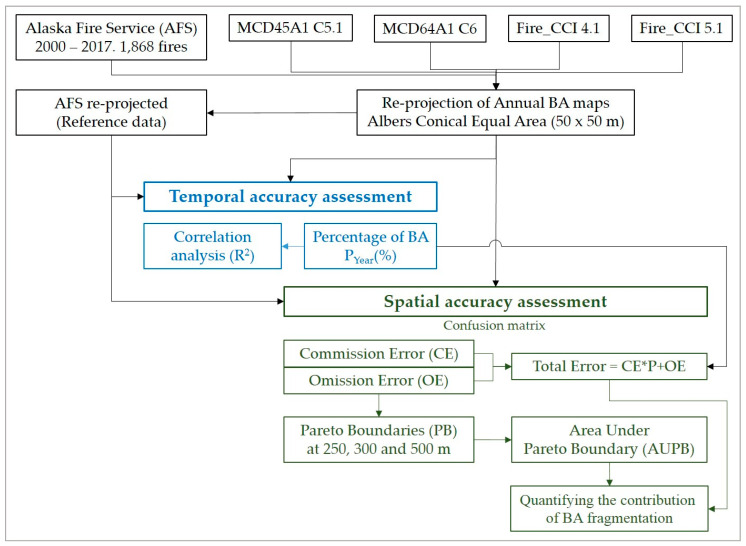
Flowchart followed for the accuracy assessment of standard BA products.

To validate the burned area products, an accuracy assessment was performed against the AFS reference data for those periods when each of the products was available (Table 1). The AFS perimeters used in this study are derived primarily from satellite images with spatial resolutions of less than 30 m, including the Landsat and Sentinel-2 missions, the latter since 2016. Figure 5 shows a description of the workflow of this assessment exercise carried out on an annual basis. All annual burned area map time series were delimited to the study region with 50 m × 50 m pixels in the same projection as the reference data. Each pixel therefore represents an area of 0.25 ha. Pixels were labeled 0 (Not Burned) or 1 (Burned). This simplified the calculation of the total annual burned area (in hectares) of each product, which was obtained by multiplying the number of pixels labeled as 1 (Burned) by 0.25.

To assess the temporal accuracy, the percentage of annual burned area for each product (P_Year_(%)) was calculated with respect to the reference set (Equation (1)).

(1) $$P_{\mathrm{Year}}\left(\%\right)=\frac{\sum_{i=1}^{n}{\mathrm{BAP}}_{\mathrm{Year}}\left(i\right)}{\sum_{i=1}^{n}{\mathrm{BAR}}_{\mathrm{Year}}\left(i\right)}\times 100$$

where BAP_Year_(i) is the value of the pixel i (0: Not Burned; 1: Burned) for the indicated year of BA product; BAR_Year_(i) is the value of the same pixel i for the reference data; and n is the total number of pixels in each burned area map.

For each annual burned area percentage distribution, a centrality measure (total value for the entire period), calculated as the weighted average of the annual percentages of burned area (${\bar{P}}_{\mathrm{BAP}}$(%)) (Equation (2)), was obtained. Y_0_ and Y_E_ are the first and last years of the BA product, respectively. Scatterplots of the annual percentages for each product were constructed against the reference data to analyze the linear dispersion around the central value and to identify the extreme cases (years with estimate percentages well below or above the mean).

(2) $${\bar{P}}_{\mathrm{BAP}}\left(\%\right)=\frac{\sum_{\mathrm{Year}=Y_{0}}^{Y_{E}}\left(\sum_{i=1}^{n}{\mathrm{BAP}}_{\;\mathrm{Year}}\left(i\right)\right)}{\sum_{\mathrm{Year}=Y_{0}}^{Y_{E}}\left(\sum_{i=1}^{n}{\mathrm{BAR}}_{\;\mathrm{Year}}\left(i\right)\right)}\times 100$$

Subsequently, a plot was constructed of the annual burned area temporal distribution of each product and that of the reference data. Correlation analysis of each time series was performed with the reference data, calculating the coefficient of determination R^2^ (the square of Pearson’s correlation coefficient).

The spatial accuracy assessment for each annual BA map employed the confusion matrix method, which is commonly used to validate thematic maps [39]. Table 2 shows the confusion matrix for a pixel-level thematic classification with two classes (Burned and Non-Burned). The independent reference information (AFS) is located in the matrix columns, and the burned area map data for each product in the rows. The diagonal elements are the correctly classified data (true Burned and true Non-Burned). The other cells indicate commission errors (CE), i.e., pixels classified as Burned that are not actually burned (false burned), or omission errors (OE), pixels that are actually burned but that have been classified as Non-Burned (false non-burned) [40]. Other commonly used metrics that can be derived from the confusion matrix are Overall Accuracy (OA) (the percentage of correctly classified pixels), Sensibility (S) (or producer’s accuracy) which calculates the rate of true burned pixels (the proportion of burned pixels that were correctly identified) and Specificity (Sp), or the rate of true Non-Burned pixels (the proportion of properly identified Non-Burned pixels) (Table 2).

The OA and Sp are metrics that can create a false sense of correctness in classified maps, especially in this study, due to the asymmetry between the two classes considered (the Non-Burned class is the majority and its success rate would be very high). On the other hand, S is related to the omission error (S = 1 − OE), so only commission and omission errors of the Burned class were considered. The two errors are not comparable since, although they both represent percentages of the pixels labeled as Burned, in one case they are Burned concerning the classified map (CE) and in the other case for the reference map (OE). Therefore, for the total error (TE) calculation, the weighted sum of both errors was considered, taking into account the percentage (P) of BA identified by the classified map, according to Equation (3).

(3) $$\mathrm{Total}\;\mathrm{Error}\;\left(\mathrm{ha}\right)=\mathrm{CE}\times \mathrm{BAP}+\mathrm{OE}\times \mathrm{BAR}=\left(\mathrm{CE}\times P+\mathrm{OE}\right)\times \mathrm{BAR}\;\mathrm{Total}\;\mathrm{Error}\;\left(\%\right)=\frac{\mathrm{Total}\;\mathrm{Error}\;\left(\mathrm{ha}\right)}{\mathrm{BAR}}=\mathrm{CE}\times P+\mathrm{OE}$$

The annual distribution of OE and CE for each burned area product was calculated, as well as their average values (the totals of all years considered). To identify extreme values, CE scatterplots were constructed against the annual OE of each product, and the annual deviations from the average values were analyzed. Likewise, to analyze the possible relationship between the spatial accuracy and the annual burned area, scatterplots of commission errors and omission errors were constructed for each BA product, against the annual burned reference area.

**Figure 6:**
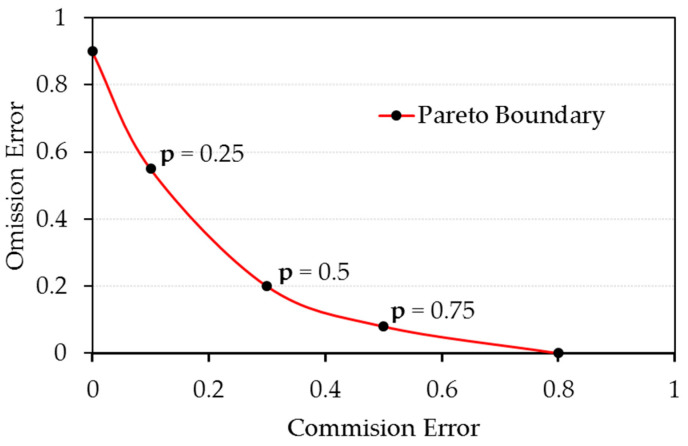
Pareto boundary for a binary classification (Burned/Non-Burned). *p* represents the minimum percentage of burned area to classify a mixed pixel as Burned.

To determine whether fragmentation of the burned areas limits the spatial accuracy of the BA products, PB were constructed at 250, 300 and 500 m spatial resolutions [41]. To obtain each PB, the AFS reference map was used, with a high spatial resolution (50 m × 50 m) per pixel. Using a pixel spatial aggregation process, the reference data was resized to maps of 250, 300 and 500 m, where the value of each pixel contained the percentage of burned area. If one of these mixed pixels is classified as Burned, in a strict binary classification, a commission error equal to a 1-pixel value is being made. However, if it is classified as Non-Burned, an omission error equal to the pixel value is produced. The final decision on how it is classified depends on a p parameter selected in the range [0, 1], which sets the minimum threshold for assigning a mixed pixel to the Burned class. Then, for each degraded map, the CE and OE pairs, obtained by varying this parameter between 0 and 1 in equidistant steps, were calculated. The set of pairs {(CE_i_, OE_i_)} resulting from this process is the PB (Figure 6). The PB represents the lowest possible errors, obtained in a strict binary classification. These errors are attributable exclusively to the fragmentation of referenced burned areas that occur in mixed pixels when the spatial resolution of the data decreases. All points on the PB represent ideal classifications for a given spatial resolution. The PB further demonstrates that minimizing the commission and omission errors simultaneously is contradictory: a classification with minimum commission errors would mean greater failure of omission and, conversely, minimizing the OE would increase the CE.

To assess the impact of the PB on the accuracy of a BA classified map, the area enclosed by it (the Area Under the Pareto Boundary, AUPB) was calculated, by analogy with the area of the ROC (Receiver Operating Characteristic) curve used in other burned area studies using machine learning or data mining [42,43,44,45,46,47,48,49,50]. The ROC curve is a probability curve constructed from sensibility and 1-specificity pairs {(S_i_,1-Sp_i_)}, obtained using a procedure similar to that of the PB, and the area enclosed by it, the Area Under the ROC Curve (AUC), is interpreted as a measure of the separability between the two classes considered from the selected **p** parameter. Similarly, a larger area enclosed by the PB implies greater fragmentation of burned areas to that spatial resolution, and therefore a greater contribution to commission and omission errors by the mixed pixels. 

The estimate of the area enclosed by the PB was calculated by applying the trapezoidal rule and by considering CE as the independent variable Equation (4) and OE as the dependent variable.

(4) $$\mathrm{Area}=\overset{1}{\underset{0}{\int }}\mathrm{OE}\left(\mathrm{CE}\right)\times \mathrm{dCE}≅\underset{i}{\sum }\frac{{\mathrm{OE}}_{i+1}+{\mathrm{OE}}_{i}}{2}\times \left({\mathrm{CE}}_{i+1}-{\mathrm{CE}}_{i}\right)$$

where OE(CE) is the curve representing the PB, defined from the point pairs {(CE_i_,OE_i_)}.

Finally, to quantify the contribution of burned area fragmentation in the errors of the annual BA maps, linear regression models were constructed that related the annual areas under the PB curve to the total annual errors of each area burned product to its corresponding spatial resolution.

## 3. Results

### 3.1. Temporal Accuracy

Table 3 presents the annual BA percentages detected by the different products based on the AFS reference data. The years in which these products are available within the study period (2000–2017) are indicated. The total values (average percentage) for the entire period under consideration are also included. All the products underestimated the BA total for each of the years analyzed. Fire_CCI 5.1 presented the best overall results (65.89%), almost double that of version 4.1 (34.53%). Similarly, the MCD64A1 product showed estimates in the same order as Fire_CCI 5.1, although slightly lower (63.22%), an improvement which is more than double that of the previous product, MCD45A1 (28.11%). 

Overall, there was an improvement in the BA estimate with the new versions for all the common years of the time series, with only two exceptions: in 2006, when version 4.1 of Fire_CCI gave a better annual burned area estimate than version 5.1 (52.42% vs 45.83%), and in 2000, when MCD45A1 C5.1 did the same with respect to MCD64A1 C6 (6.79% vs 0.0%). MCD64A1 also presented a low percentage in the burned area estimate in 2006 (29.6%), 2010 (38.51%), 2011 (12.71%) and 2013 (47.23%), well below the average value. On the other hand, Fire_CCI 5.1 showed greater stability in annual burned area estimates, and, apart from the particular case of 2001, it was only in 2006 that it showed a percentage less than 50% (45.83%). The years 2001 and 2006 recorded a smaller amount of burned area (0.09 and 0.11 million ha), so their contribution to the average values for the entire period was very small. However, it was also observed (Table 3) that there were years with low fire activity even though the estimate percentages in both products were higher. For example, in 2008, when 0.04 million ha were burned, MCD64A1 and Fire_CCI 5.1 gave above-average values for the estimate percentages of 67.49% and 79.43%, respectively.

**Figure 7:**
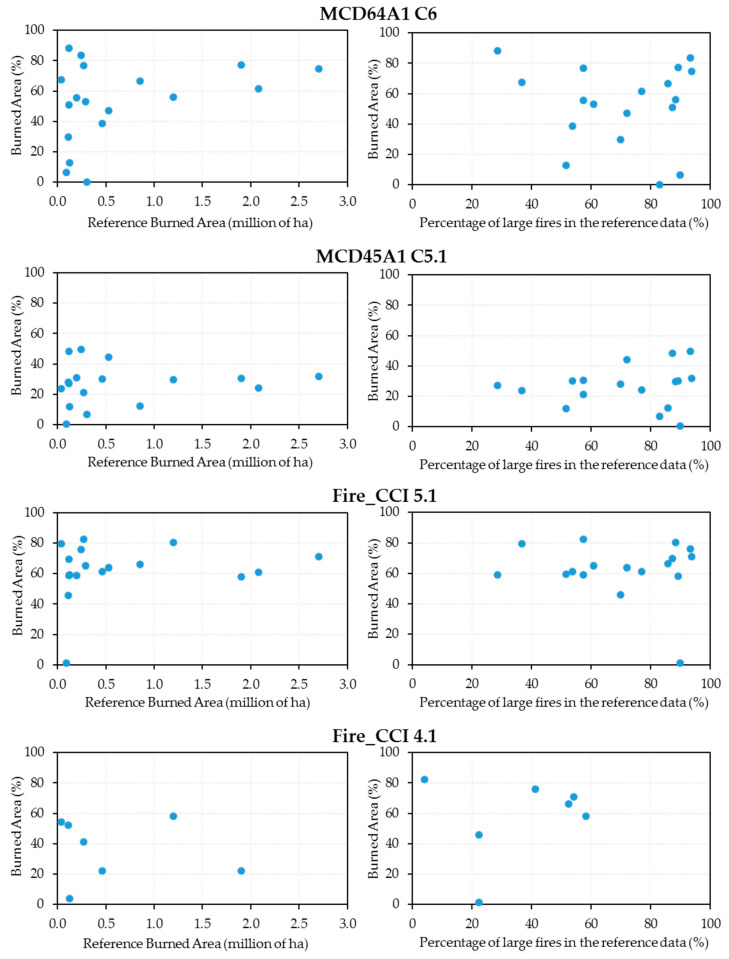
Scatterplots of the annual burned area percentages for the four products analyzed against the annual reference burned area (left) and versus the percentage of burned area of ≥10.000 ha fires (right).

Figure 7 shows the scatterplots for the percentage of annual burned area detected by each of the four products analyzed against the annual burned area recorded by AFS (in millions of hectares). The right panel in Figure 7 represents the same percentages of annual burned area versus the percentage of large fires (above 10,000 ha) in the reference data. For MCD64A1, the highest percentages of burned area (83.36% and 88.14%, respectively) were detected in 2003 and 2012, but the percentages corresponding to large fires are disparate (73.6% and 28.5%, respectively). Similarly, for Fire_CCI 5.1, 2007, 2008 and 2009 had the highest detection rates (82.46%, 79.43% and 80.36%, respectively) with very different large fire percentages (57.4%, 36.7% and 88.4%, respectively). In both products, the year 2001 stands out, when there was a very low detection rates (1.23% and 6.64%) despite the percentage of large fires being 89.9%.

**Figure 8:**
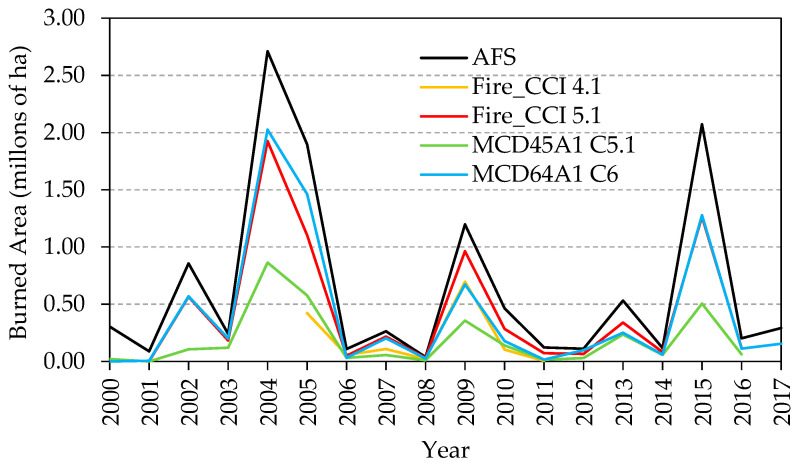
Temporal distribution of the annual burned area estimation for the Fire_CCI 4.1, Fire_CCI 5.1, MCD45A1 C5.1 and MCD64A1 C6 products along with the AFS reference data.

To analyze the temporal correlation of the reference data, the temporal distribution of the annual BA for the different products are represented along with the reference data (Figure 8). All products conformed to the temporal pattern of the AFS reference data, with determination coefficients of 0.991 (Fire_CCI 5.1), 0.987 (MCD64A1), 0.973 (MCD45A1) and 0.817 (Fire_CCI4.1).

### 3.2. Spatial Accuracy

Table 4 shows the results of the main metrics derived from the confusion matrix (OE, CE and TE) for each year and for each BA product, together with the average for all years. Older versions of Fire_CCI and MCDs had the lowest average CE (5.9% and 6.6%, respectively). However, the lowest average OE (39.0% and 48.0%, respectively) are for the new versions, as are the lower average TE (43.9% and 59.3%, respectively).

On average, Fire_CCI 5.1 had lower commission and omission errors than the MCD64A1 product, and therefore higher positional accuracy. Similar to the BA estimates, a high annual variability was observed in both types of errors with respect to the average values: for the Fire_CCI product, the annual commission error fluctuated in the interval [4.1%, 30.3%] and the omission [24.7%, 99.1%], while, for the MCD64A1 product, the CE fluctuated in the interval [0%, 57.3%] and the OE [34.5%, 100%].

While the new Fire_CCI product version significantly improves the dispersion of both errors around their central values compared to the previous version, the same does not appear to be the case with the MCD64A1 product, where several years are identified with annual CE above 30%: 2001 (100%), 2003 (38.6%), 2006 (42.4%), 2007 (31.6%), 2008 (57.3%) and 2012 (51.6%). For Fire_CCI 5.1, the 20% threshold for annual CE was only exceeded in three years: 2001 (30.3%), 2008 (29.2%) and 2011 (23.0%). In these years, the reference burned area was below 0.3 million ha, so its contribution to average CE was very small. The years with the highest amount of burned area recorded, which contribute the most to the average error, showed lower CE for both products: 2004 (17.4% and 6.4%), 2005 (15.1% and 8.9%) and 2015 (15.5% and 4.1%). For omission errors, MCD64A1 reported errors of more than 70% for five years of low fire activity: 2000 (100%), 2001 (100%), 2006 (83%), 2008 (71.2%) and 2011 (90.1%). The last version of Fire_CCI only exceeded 60% in two years: 2001 (99.1%) and 2006 (60.9%). Both products demonstrated a noticeable improvement in OE compared to previous versions, but worse CE.

The Fire_CCI product showed the best CE, and only in three years was the 20% threshold exceeded: 2001 (30.3%), 2008 (29.2%) and 2011 (23.0%). These were years when the reference burned area was among the lowest of the entire series (below 125.000 ha). Similarly, the OE were also better; they only exceeded 50% in the three years 2001 (99.1%), 2006 (60.9%) and 2011 (54.3%), when the burned area was also among the lowest values of the series. The variability of both errors around their central values was very low in years with fire activity above 0.5 million ha ([0.041, 0.095] for the commission and [0.247, 0.470] for the omission), but increased in years with fire activity below 0.5 million ha ([0.065, 0.290] for the commission and [0.303, 0.609] for the omission). For the MCD64A1 product, the variability around the average commission and omission errors was also less with increased annual burned area ([0.094, 0.219] for the commission and [0.345, 0.491] for the omission); however, for years with low fire activity, the variability was much greater than for the Fire_CCI product ([0.111, 0.573] for the commission and [0.476, 0.901] for the omission).

**Figure 9:**
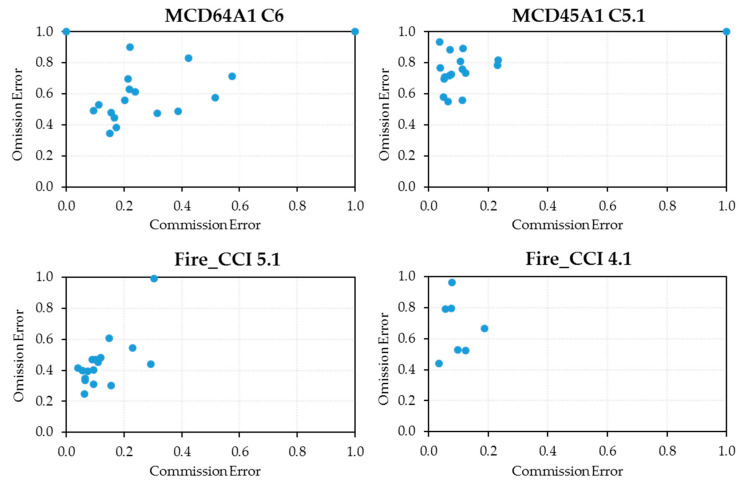
Scatterplots of annual omission errors versus annual commission errors for each of the four burned area products analyzed.

Figure 9 shows the scatterplots of annual omission errors versus annual commission errors for each of the products under consideration. Extreme values stand out, with a TE of around 100%, corresponding to the years 2000 and 2001. A qualitative analysis of these charts shows that, in general, new versions of both products have shifted the point cloud significantly down (less omission error) but have also shifted it slightly to the right (slightly increasing the commission error). The higher point cloud concentration of the Fire_CCI 5.1 product also indicates lower CE and OE variability.

**Figure 10:**
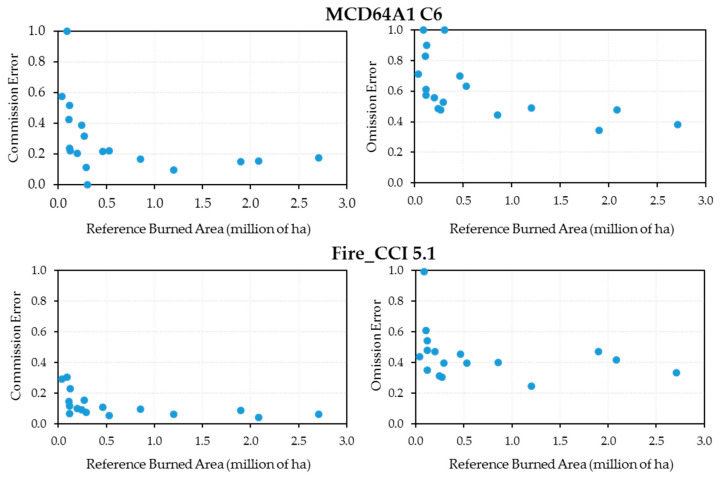
Scatterplot for the annual commission and omission errors versus the annual reference burned area.

Figure 10 shows the annual commission and omission error scatterplots against the annual reference burned area for the latest versions of the MCD64A1 and Fire_CCI products. Both errors fluctuated strongly when the annual reference burned area was below 0.5 million ha, whereas they stabilized around their average for higher values. The fluctuations were higher for the MCD64A1 C6 product than for the Fire_CCI 5.1 product, in line with higher average values.

### 3.3. Pareto Boundaries

**Figure 11:**
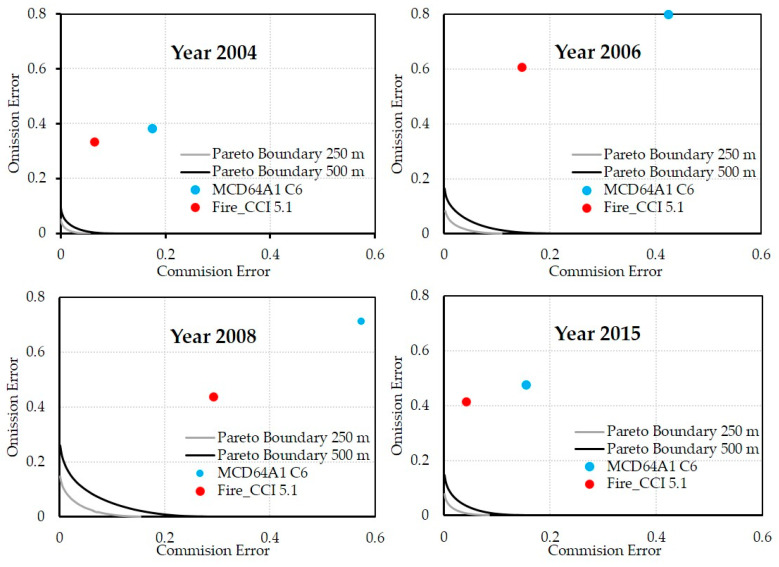
Pareto boundaries at 250 and 500 m and annual CE and OE for the MCD64A1 and Fire_CCI 5.1 products for 2004, 2006, 2008 and 2015.

The Pareto boundaries were constructed at 250/300/500 m for all years in the study period. Figure 11 shows the Pareto boundaries at 250 and 500 m for 2004, 2006, 2008 and 2015, as well as the annual CE and OE for the MCD64A1 and Fire_CCI 5.1 products. Years 2006 and 2008 were selected because both have the biggest errors in both products. In addition, two years, 2004 and 2015, with good behavior were included, to provide a visual comparison of the PB. One can observe that in 2006 and 2008 the distance from the annual pair of errors (EC, EO) to the PB is much larger than in 2004 and 2015. It can also be seen that the PB are more separated from the Cartesian axes in those years. This indicates a greater fragmentation of the burned areas when the spatial resolution is degraded, which contributes, in part, to the errors of the corresponding annual map.

To quantify the impact of the PB on the annual maps of the different BA products, the annual and total AUPB values were calculated at spatial resolutions of 250/300/500 m (Table 5). On average, the AUPB value at 500 m triples the 250 m value, in addition to having greater annual variability: [0.0019–0.0146] versus [0.0007–0.0048]. The year 2008 shows the highest AUPB values for all spatial resolutions.

**Figure 12:**
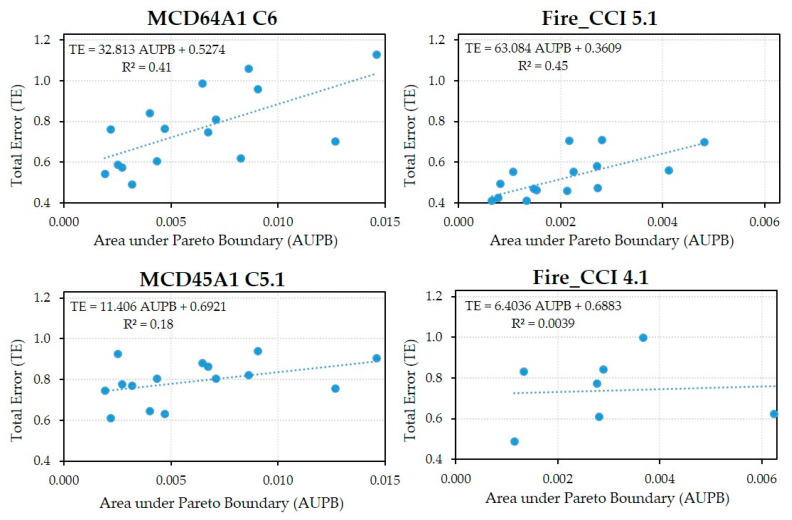
Scatterplots of the total annual TE errors (weighted sum of the annual commission and omission errors) versus the area enclosed by the annual Pareto boundary. Data for the years 2000 and 2001 were not included, as there were incomplete data from the MODIS sensor.

Finally, Figure 12 shows the annual TE scatterplots for each product versus the annual PB area corresponding to their spatial resolution. A linear regression model was constructed for each BA product. The R^2^ values are very low for the older versions of the BA products (0.00 and 0.18, respectively), but increase markedly in the new versions. Fire_CCI 5.1 has a slightly higher value than MCD64A1 C6 (0.45 vs. 0.41) and also a linear regression line slope that is almost double (63.1 vs. 32.8).

## 4. Discussion

The evaluation and validation of global BA products derived from satellite imagery require a set of reliable, independent and representative reference fire perimeters that cover as long a period as possible. It is necessary to understand the uncertainty of these products before incorporating them as input data into global carbon, vegetation or climate models, as well as for the management of all the wildfire phases. Numerous prior studies have evaluated the behavior of the MODIS sensor-derived products analyzed in this study, both as part of ESA’s Fire Climate Change Initiative Project and in the different versions [13,51,52,53,54,55,56,57,58] of the MODIS Direct Broadcast Monthly Burned Area Product. However, many of these works construct reference fire perimeters using images from better spatial resolution sensors such as Landsat TM/ETM or Sentinel-2, and which are limited to short time periods, usually one or several years, due to the difficulty in creating larger reference sets. This work presents an independent intercomparison exercise on the accuracy of the two main global BA products derived from the MODIS sensor covering an extensive boreal region over 18 years, containing all the AFS-registered fire perimeters. To the best of our knowledge, there have been no previous intercomparison studies of these four products over such a long period.

In relation to the temporal accuracy (Figure 8), and except for the Fire_CCI 4.1 product, which has the shortest time series, the BA products analyzed conform to the time pattern of the reference data with determination coefficients above 0.97, and with results similar to those found by other authors in different ecosystems. Thus, for example, Turco et al. [56] reported high determination coefficients of 0.96 and 0.97 in the monthly BA estimates for Fire_CCI 5.1 and MCD64A1 C6 when compared to the reference dataset from the European Forest Fire Information System (EFFIS) that includes burned area data for some European countries in the Mediterranean basin (Portugal, Spain, Southern France and Greece). However, all the BA products analyzed underestimated the annual burned area (Table 3) with higher percentages than those obtained by other authors for regions other than Alaska’s boreal forest. Turco et al. [56] obtained an underestimation of only 14% for the Fire_CCI 5.1 product with the EFFIS reference set. Campagnolo et al. [57] found a 28% underestimation for the MCD64A1 product using a reference set of more than 100 fire perimeters (1.24 Mkm^2^) derived from Landsat TM/ETM+ images distributed around the world for 2008 and previously constructed by Padilla et al. [54].

From the results intercomparison determined for the two new versions (Table 3), one can show that MCD64A1 provided better BA estimates than Fire_CCI in some years, despite having a lower spatial resolution (500 vs. 250 m), even in the years with the largest BA, which are the ones that contributed more to the average values (2004, 2005 and 2015); nevertheless, it performed relatively worse in other years (2009, 2010 and 2011). For the MCD64A1 and Fire_CCI 5.1 products, in the years with the highest amount of BA (over 1 million ha), the annual estimate percentages tended to stabilize around the average value with lower variability (Figure 7). One should bear in mind that it is precisely these years that contribute most to the overall average throughout the study period. Conversely, the years with the least amount of BA (below 0.5 million ha) had greater variability; this was less pronounced in the Fire_CCI 5.1 product than in MCD64A1, probably due to its better spatial resolution (250 vs. 500 m).

With regard to spatial accuracy (Table 4), a global analysis showed that the older versions, Fire_CCI 4.1 and MCD45A1 C5.1, had the lowest commission errors (5.9% and 6.6%, respectively) but the omission errors were very high (67.5% and 73.7%, respectively). The new versions, Fire_CCI 5.1 and MCD64A1 C6, had significantly reduced OE (up to 39.0% and 48.0% respectively), even at the cost of slightly worse CE (7.5% and 17.8%, respectively). As the percentage of BA of all products was below 100% (they underestimated the total BA), the weighted sum or TE tended to give a lower weight to the CE than to the OE, favoring the algorithms that reduce the omission error even at the cost of increasing the commission errors. This low imbalance between omission and commission errors (along with the sharp drop in OE) translates into a better BA estimate. These results are comparable to those obtained by other authors using other reference sets [51,58]. In a recent validation of Fire_CCI 5.1 using 1200 global samples over the 2003–2014 period, Lizundia-Loiola et al. [58] obtained values of CE = 54.4% and OE = 67.1%. In the Stage 3 validation of MCD64A1 C6 using 558 pairs of Landsat images from 2014 and 2015, Boschetti et al. [51] obtained values of CE = 40.2% and OE = 72.6% on a global scale, which improved significantly for boreal forest biomass (CE = 23.9% and OE = 27.0%). The OE from this latter study, which was specific to the boreal region, differs significantly from that obtained in the present work (48.0%) for the Alaska region; we understand that this may be due to the small reference dataset used by Boschetti et al. [51] for the entire boreal region. In contrast, our study used all the burned area perimeters recorded in the 2000–2017 period.

The PB analysis (Figure 11) allowed us to partially explain the annual variability in the commission/omission errors of the BA products. The average AUPB values (Table 5) reflect an increase as the spatial resolution worsens, indicating that there is a higher percentage of commission and omission errors attributable to the data’s low spatial resolution. Again, there was high variability in the annual values relative to the average values, attributable to the fragmentation of the burned areas. This high accuracy variability attributable to landscape fragmentation was also reported by Rodrigues et al. [59], in the accuracy assessment of MCD64A1 C6 in the Brazilian Cerrado vs. Landsat perimeters for the 2011–2019 period. Rodrigues et al. [59] found that, in the northern Cerrado, which had larger areas affected by fire, the MCD64A1 C6 performance was significantly higher than in the southern Cerrado area, where a more fragmented landscape and smaller patches of fire predominated.

The linear regression analysis between the TE and the AUPB found an upward linear trend for the new versions of the BA products; this partially justifies the high commission and omission errors encountered in some years. However, it is also apparent that in some years (e.g., 2016), the high level of fire fragmentation had little influence on the TE. Conversely, in years with low levels of fragmentation (e.g., 2002 and 2005 for Fire_CCI 5.1 and 2003 and 2014 for MCD64A1 C6), the TEs were significant. For these years, the observations were limited by other factors such as the poor behavior of the detection algorithms, the low severity of burned areas or certain environmental conditions; as indicated by Loboda et al. [60], these factors might explain such errors. The more precise linear fit of the new versions compared to the old ones, with R^2^ values close to 0.5 and a higher slope, reflects greater TE sensitivity (CE and OE) to burned area fragmentation.

Of all the years analyzed, and except for the years 2000 and 2001 (years with incomplete data from the MODIS sensor), there are two years in which the latest versions of MCD64A1 and Fire_CCI performed poorly. The year 2006 showed low detection rates (29.6% and 45.83%, respectively), high omission values (83.9% and 60.9%) and high commission values (29.6% and 14.7%). The burned area recorded (0.11 million ha) was below the annual average, as was the same percentage corresponding to large fires (69.82%), which was also below the annual average, while the PB areas at 250 (0.002257) and 500 m (0.006480) were almost double the average values at these resolutions. Similarly, in 2008, the year with the least amount of burned area (0.04 million ha) and the lowest percentage attributable to large fires (36.65%), there were high commission and omission error values (57.3% and 71.2% for MCD64A1 and 29.2% and 43.8% for Fire_CCI) but a large percentage of burned area detected (67.49% and 79.43%, respectively). For that year, the area enclosed by the PB at 250/500 m was the highest, so a significant part of the spatial errors was due to the data’s low spatial resolution.

## 5. Conclusions

An independent and detailed study was carried out to evaluate the spatiotemporal accuracy of the latest versions (along with the previous versions) of the two main global scale BA products derived from MODIS-sensor satellite images, here restricted to the Alaskan boreal region. As reference data, we used all the polygons of the BA recorded by AFS over the study period. In addition, a detailed 50 m × 50 m pixel analysis of the accuracy of each BA product was performed. Fire_CCI 5.1 and MCD64A1 C6 presented significant improvements over their previous versions in terms of BA estimation. Improvements were achieved by reducing the imbalance between commission and omission errors and, especially, by greatly reducing omission errors even at the expense of worsening commission errors. Both products currently produce similar BA estimation percentages, although the positional accuracy of Fire_CCI is better than that of MCD64A1, which is in line with its higher spatial resolution (250 vs. 500 m). Fire_CCI 5.1 would be the option chosen for users who, through geoinformatics analysis techniques, use BA products for forest fire management. For those users who are studying the increase in greenhouse gas concentrations or the change in the chemical composition of the atmosphere due to fire emissions, any of the latest versions of the products analyzed could be suitable, but always taking into account the errors of omission (over 40%) in both cases.

The high variability in annual results is noteworthy, both in the percentages of BA detection and in the omission and commission errors, which puts into question much accuracy assessment work that uses limited spatiotemporal reference data. It would be recommendable to use all possible reference data when available.

The yearly analysis of burned-area fragmentation across the corresponding Pareto boundaries has allowed us to establish a quantitative measure of the same (AUPB), which relates to the total errors (weighted sum of commission and omission errors) obtained for the latest versions of the Fire_CCI and MCD64A1 products. The results from this work could be extrapolated to other boreal regions that do not have such accurate reference datasets as are available in the northern regions of North America.

## Figures and Tables

**Table 1:** Burned area products.

Product	Time Span	Sensor	Method	Spatial Resolution	Algorithm References
Fire_CCI 4.1	2005–2011	MERIS + Terra MODIS	Reflectance + hotspots	300 m	[33]
Fire_CCI 5.1	2001–today	Terra MODIS	Reflectance + hotspots	250 m	[12]
MCD45A1 C5.1	2000–2016	Terra/Aqua MODIS	Reflectance	500 m	[34,35,36]
MCD64A1 C6	2000–today	Terra/Aqua MODIS	Reflectance + hotspots	500 m	[13]

**Table 2:** Confusion matrix of a binary classification of burned area and the main metrics derived from it. OA, overall accuracy; S, sensibility or producer’s accuracy; Sp, specificity; CE, commission error; OE, omission error.

		Reference Data		
		Burned	Non-Burned	Total
Classified Data	Burned	n_11_	n_12_	n_1c_
	Non-Burned	n_21_	n_22_	n_2c_
	Total	n_1r_	n_2r_	n
$\mathrm{OA}=\frac{n_{11}+n_{22}}{n}\;S=\frac{n_{11}}{n_{1r}}\;\mathrm{Sp}=\frac{n_{22}}{n_{1r}}\;$				
$\mathrm{CE}=\frac{n_{12}}{n_{1c}}\;\mathrm{OE}=\frac{n_{21}}{n_{1r}}$				

**Table 3:** Annual Reference BA (AFS, Fort Wainwright, AK, USA) and estimates (%) of the four BA products Fire_CCI 4.1, Fire_CCI 5.1, MCD45A1 C5.1 and MCD64A1 C6. In the last row, the value for the AFS (ha) column is the sum of BA for all the years and the percent of BA detected for all years for the rest of the columns.

Year	AFS (ha)	Fire_CCI 4.1 (%)	Fire_CCI 5.1 (%)	MCD45A1 C5.1 (%)	MCD64A1 C6 (%)
2000	304,631.50			6.79	0.00
2001	88,658.25		1.23	0.40	6.64
2002	856,081.50		66.06	12.30	66.44
2003	241,061.25		75.92	49.76	83.36
2004	2,712,368.00		71.02	31.88	74.73
2005	1,896,684.75	22.23	58.17	30.40	77.11
2006	108,509.00	52.42	45.83	28.18	29.60
2007	263,894.00	41.26	82.46	21.12	76.64
2008	39,164.50	54.20	79.43	23.74	67.49
2009	1,198,139.50	58.17	80.36	29.78	56.17
2010	46,494.00	22.22	61.34	30.19	38.51
2011	122,486.75	3.91	59.29	11.85	12.71
2012	111,290.25		58.97	27.14	88.14
2013	532,279.00		63.72	44.37	47.23
2014	117,193.00		69.59	48.26	51.04
2015	2,073,041.25		61.02	24.38	61.69
2016	201,944.75		58.92	30.75	55.52
2017	292,026.50		65.17		52.91
All years	11,623,847.75	34.53	65.89	28.11	63.22

**Table 4:** Commission (CE), Omission (OE) and Total (TE) errors of the Fire_CCI 4.1, Fire_CCI 5.1, MCD45A1 C5.1 and MCD64A1 C6 burned area products for each year and the weighted average errors (the weights are the annual percentage of BA with respect to total BA) for all years.

Year	Fire_CCI 4.1			FIRE_CCI 5.1			MCD45A1 C5.1			MCD64A1 C6		
	CE	OE	TE	CE	OE	TE	CE	OE	TE	CE	OE	TE
2000							0.037	0.935	0.938	0.000	1.000	1.000
2001				0.303	0.991	0.995	1.000	1.000	1.004	1.000	1.000	1.066
2002				0.095	0.402	0.465	0.071	0.886	0.895	0.166	0.446	0.556
2003				0.093	0.311	0.382	0.113	0.558	0.614	0.386	0.488	0.810
2004				0.064	0.335	0.380	0.053	0.698	0.715	0.174	0.383	0.513
2005	0.056	0.790	0.802	0.089	0.470	0.522	0.069	0.717	0.738	0.151	0.345	0.461
2006	0.097	0.527	0.578	0.147	0.609	0.676	0.232	0.784	0.849	0.424	0.830	0.956
2007	0.188	0.665	0.743	0.155	0.303	0.431	0.107	0.811	0.834	0.316	0.476	0.718
2008	0.124	0.525	0.592	0.292	0.438	0.670	0.234	0.818	0.874	0.573	0.712	1.099
2009	0.034	0.438	0.458	0.062	0.247	0.297	0.075	0.725	0.747	0.094	0.491	0.544
2010	0.077	0.795	0.812	0.110	0.454	0.521	0.125	0.736	0.774	0.214	0.697	0.779
2011	0.079	0.964	0.967	0.230	0.543	0.679	0.116	0.895	0.909	0.221	0.901	0.929
2012				0.118	0.480	0.550	0.113	0.759	0.790	0.516	0.574	1.029
2013				0.055	0.398	0.433	0.049	0.578	0.600	0.219	0.631	0.734
2014				0.065	0.350	0.395	0.065	0.549	0.580	0.238	0.611	0.732
2015				0.041	0.415	0.440	0.038	0.766	0.775	0.155	0.478	0.574
2016				0.101	0.470	0.530	0.055	0.709	0.726	0.203	0.558	0.671
2017				0.073	0.396	0.444				0.111	0.529	0.588
All years	0.059	0.675	0.695	0.075	0.390	0.439	0.066	0.737	0.756	0.178	0.480	0.593

**Table 5:** Areas (×10^−3^) enclosed by the annual Pareto Boundaries (AUPB) at different resolutions and the weighted average value for all years.

Year	Spatial Resolution		
	250 m	300 m	500 m
2000			4.885
2001	1.670		4.940
2002	0.824		2.527
2003	1.341		4026
2004	0.656		1.909
2005	1.066	1.333	3.191
2006	2.171	2.810	6.480
2007	2.125	2.767	6.724
2008	4.818	6.249	14.610
2009	0.931	1.149	2.714
2010	2.257	2.891	7.106
2011	2.810	3.680	9.056
2012	2.715		8.642
2013	1.534		4.723
2014	0.778		2.192
2015	1.470		4.356
2016	4.120		12.685
2017	2.733		8.253
All years	1.195	1.625	3.612
